# Correction: *Ooencyrtus pitosina* (Hymenoptera: Encyrtidae)–A natural enemy of Samoan swallowtail butterfly *Papilio godeffroyi* (Lepidoptera: Papilionidae)

**DOI:** 10.1371/journal.pone.0322818

**Published:** 2025-04-22

**Authors:** Andrew Polaszek, John S. Noyes, Elena B. Lugli, Mark A. Schmaedick, Robert W. Peck, Paul C. Banko, Lucian Fusu

## Notice of Republication

This article was republished on March 28, 2025, to correct an error in which the figures were incorrectly switched. Please download the article again to view the corrected version. Both the originally published, uncorrected article and the republished, corrected article are provided here for reference.

## Supporting information

S1 File Originally published, uncorrected article. (PDF)

S2 File Republished, corrected article. (PDF)
