# Supplementary material for: Correction: Ooencyrtus pitosina (Hymenoptera: Encyrtidae)–A natural enemy of Samoan swallowtail butterfly Papilio godeffroyi (Lepidoptera: Papilionidae)
Source: PLoS One. 2025 Apr 22;20(4):e0322818. doi: 10.1371/journal.pone.0322818 (PMC12013940; doi:10.1371/journal.pone.0322818)
Supplement: S2 File — (PDF) [file pone.0322818.s002.pdf]

PLOS ONE

RESEARCH ARTICLE

Ooencyrtus pitosina (Hymenoptera: Encyrtidae)—A natural enemy of Samoan swallowtail butterfly *Papilio godeffroyi* (Lepidoptera: Papilionidae)

Andrew Polaszek[1]*, John S. Noyes[1], Elena B. Lugli[2], Mark A. Schmaedick[3], Robert W. Peck[4], Paul C. Banko[5], Lucian Fusu[6]

1 Natural History Museum, London, England, United Kingdom, **2** Core Research Laboratories, Natural History Museum, London, England, United Kingdom, **3** Division of Agriculture, Community and Natural Resources, American Samoa Community College, Pago Pago, American Samoa, **4** Hawai'i Cooperative Studies Unit, University of Hawai'i at Hilo, Hilo, Hawai'i, United States of America, **5** U.S. Geological Survey, Pacific Island Ecosystems Research Center, Kilauea Field Station, Hawai'i, United States of America, **6** Research Group in Invertebrate Diversity and Phylogenetics, Al. I. Cuza University, Faculty of Biology, Iasi, Romania

* a.polaszek@nhm.ac.uk

Check for updates

OPEN ACCESS

Citation: Polaszek A, Noyes JS, Lugli EB, Schmaedick MA, Peck RW, Banko PC, et al. (2023) *Ooencyrtus pitosina* (Hymenoptera: Encyrtidae)—A natural enemy of Samoan swallowtail butterfly *Papilio godeffroyi* (Lepidoptera: Papilionidae). PLoS ONE 18(8): e0288306. https://doi.org/10.1371/journal.pone.0288306

Editor: Ramzi Mansour, University of Carthage, TUNISIA

Received: April 8, 2023

Accepted: June 16, 2023

Published: August 9, 2023

Copyright: This is an open access article, free of all copyright, and may be freely reproduced, distributed, transmitted, modified, built upon, or otherwise used by anyone for any lawful purpose. The work is made available under the Creative Commons CC0 public domain dedication.

Data Availability Statement: All relevant data are within the manuscript and via GenBank Accession Numbers: OQ648163–OQ648167 (COI) and OQ650244–OQ650251 (28S).

Funding: AP and EBL were supported through an award from the U.S. Geological Survey (USGS contract #140G0322P0086). MAS was supported through the U.S. Department of Agriculture National Institute of Food and Agriculture Hatch Program. PCB and RWP were supported by the

Abstract

A new species of encyrtid wasp, *Ooencyrtus pitosina* Polaszek, Noyes & Fusu **sp. n.**, (Hymenoptera: Encyrtidae: Encyrtinae) is described as a gregarious parasitoid in the eggs of the endemic Samoan swallowtail butterfly *Papilio godeffroyi* (Lepidoptera: Papilionidae) in the Samoan archipelago. It is described here because it is an important natural enemy of this butterfly, and to facilitate identification for future work with this parasitoid and its host.

Introduction

The Samoan swallowtail butterfly (*Papilio godeffroyi* Semper 1866), is one of seven endemic butterfly species in the Samoan Archipelago [1, 2] and is classified as "Endangered" due to declining range, habitat, and population [3]. The butterfly, which inhabits relatively undisturbed rainforest, oviposits on *Micromelum minutum* (family Rutaceae), a small tree indigenous to the Samoan Archipelago, and distributed widely in the Indo-Pacific [4]. Nearly 140 years ago, Mathew [5] observed that the eggs of the Samoan swallowtail were attacked by an unknown parasitoid wasp. He also noted heavy parasitism of the eggs of the Fijian swallowtail butterfly, *Papilio schmeltzi* (Herrich-Schäeffer 1869), which today is classified as "Near Threatened" [6]. Of the Fijian swallowtail, he said (page 358), "These ova were terribly subject to the attacks of a minute hymenopterous parasite. Only about one in a dozen produced a larva, the remainder giving birth to three or four ichneumons, so small that they were barely visible to the naked eye." Of the Samoan swallowtail he wrote (page 362), "They were also terribly attacked by the same kind of parasite." Mathew did not report on the number of eggs he attempted to rear for either butterfly species, but his observations clearly indicated high rates of attack, possibly by the same or similar parasitoid wasp species.

Natural Resources Protection Program (NRPP), a collaboration between the USGS and the National Park Service. Additional support to PCB was provided by the USGS Wildlife Program. The funders had no role in the study design, data collection and analysis, decision to publish, or preparation of the manuscript. https://doimspp.sharepoint.com/sites/nps-biologicalresourcesdivision/SitePages/USGS-NRPP-Funding-Source.aspx?CT=1680899434511&OR=OWA-NT&CID=94d148c4-7802-4cc1-cd84-ed03461d8c38 USGS Wildlife Program https://www.usgs.gov/programs/species-management-research-program/science/wildlife-and-terrestrial-species USDS-NIFA Hatch Program https://www.nifa.usda.gov/grants/programs/capacity-grants/hatch-act-1887.

Competing interests: The authors have declared that no competing interests exist.

As part of a life history investigation of *P. godeffroyi*, conducted on Tutuila Island, American Samoa from November 2013 to June 2014 [7], we frequently observed an undescribed species of wasp, *Ooencyrtus* (Hymenoptera: Encyrtidae), emerging from field collected eggs [8]. Up to five wasps were observed emerging from a single butterfly egg. We collected *M. minutum* leaflets bearing eggs of *P. godeffroyi*, and transported them to the laboratory at American Samoa Community College (ASCC) for rearing. In the laboratory, excised leaf pieces bearing intact eggs were placed in 1-cm diameter, 3.5 cm-tall glass shell vials with plastic caps containing 0.5 cm-diameter holes covered with 100 mesh stainless wire cloth. The eggs were maintained in an incubation chamber at 27˚C with a photoperiod of 14 h light and 10 h dark until larvae or parasitoids emerged. Parasitoids were preserved in molecular grade absolute ethanol for molecular analysis.

Our observations, together with Mathew's [5] historical account, have motivated this taxonomic determination to facilitate identification in the future and to provide the formal nomenclature critical for additional work with this parasitoid and its hosts. Although we are not aware of any wasp specimens that Mathew may have collected from Fijian or Samoan swallowtail eggs, we expect that the new species we describe here may be the same, at least in the case of the Samoan swallowtail. We encourage others to identify parasitoids associated with the Fijian and other Pacific swallowtails to determine whether they are the same species as the one we describe here, which would indicate its distribution beyond the Samoan Archipelago.

Materials and methods

Specimen depositories: Abbreviations

AICF: Lucian Fusu collection, Al. I. Cuza University.

BPBM: Bernice P. Bishop Museum, Honolulu, Hawai'i, USA.

NHMUK: Natural History Museum, London, UK.

USNM: United States national Museum, Washington D.C., USA.

Morphological study

Morphological terminology and the format for the species description follow Noyes [9].

Abbreviations are as follows: AOD = largest diameter of anterior ocellus; AOL = minimum distance between posterior ocellus and anterior ocellus; EL = eye length; EW = eye width; FV = minimum width of frontovertex; FVL = length of frontovertex from occipital margin to top of antennal scrobes as seen in dorsal view; FVS = width of frontovertex a little above top of scrobes at a point where eye margin changes from being virtually straight to distinctly curved; FWL = fore wing length; FWW = fore wing width; GL = gonostylus length; HW = head width measured in facial view; HWL = hind wing length; HWW = hind wing width; MS = malar space (minimum distance between eye and mouth margin); MT = mid tibia length OCL = minimum distance between posterior ocellus and occipital margin; OL = ovipositor length; OOL = minimum distance between eye margin and adjacent posterior ocellus; POD = largest diameter of posterior ocellus; POL = minimum distance between posterior ocelli; SL = scape length; SW = scape width.

Card-mounted specimens were observed with a Leitz binocular microscope at magnifications ranging from 20× to 80×. Slide-mounted specimens were observed with a Leitz Dialux 20 microscope at magnifications ranging from 40 to 400×.

Images were generated as follows: Figs 1 and 2 Canon DSLR with 10× Mitutoyo objective, processed with Helicon Focus© stacking software with final editing in Adobe Photoshop© CC. Figs 3–8 were obtained using a Jenoptik Progres Gryphax NAOS digital camera attached to a Dialux 20 compound microscope using bright-field illumination. From 10 to 80 images, at

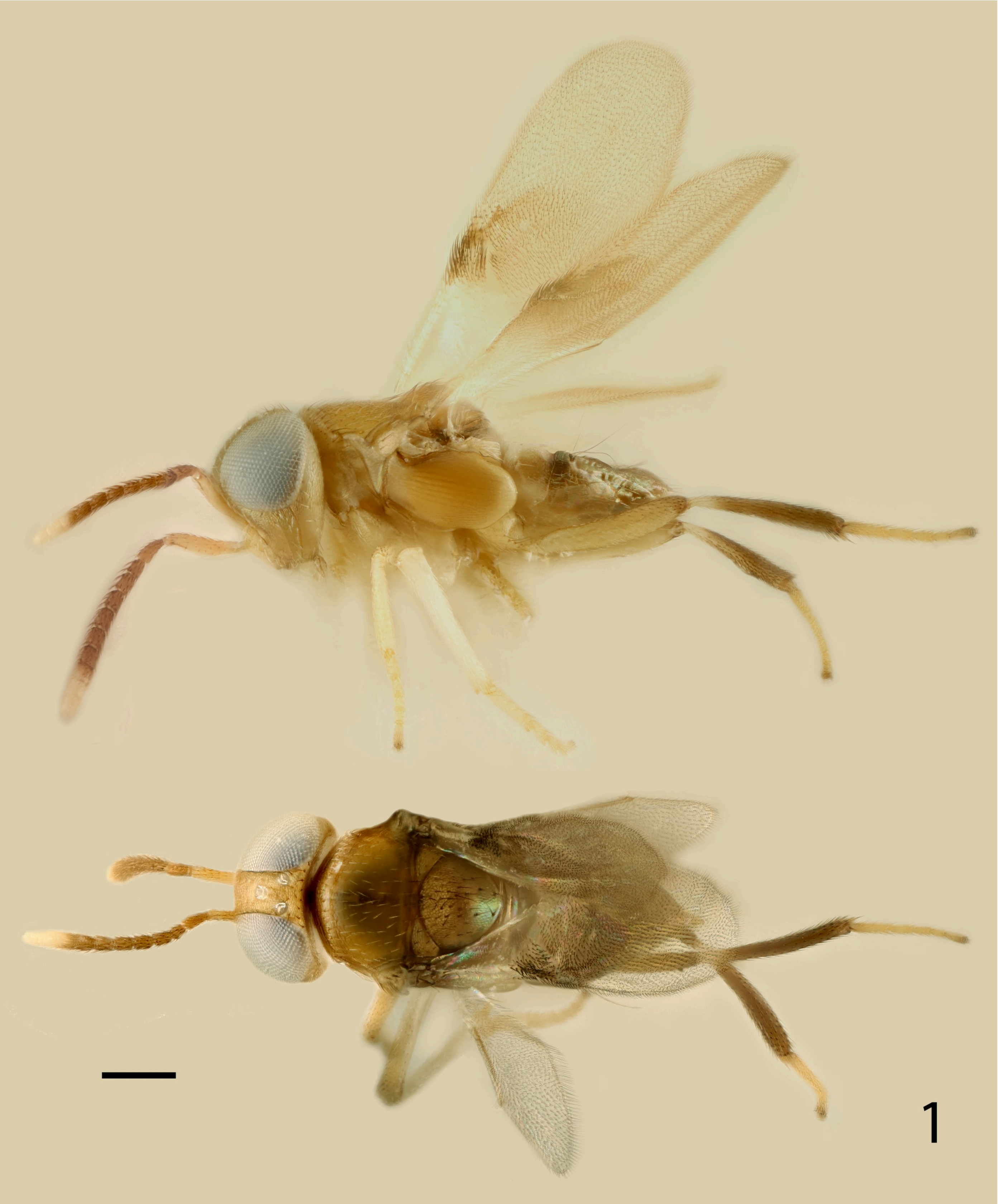

Fig 1. ***Ooencyrtus pitosina*** **holotype female dorsal and lateral habitus.**

https://doi.org/10.1371/journal.pone.0288306.g001

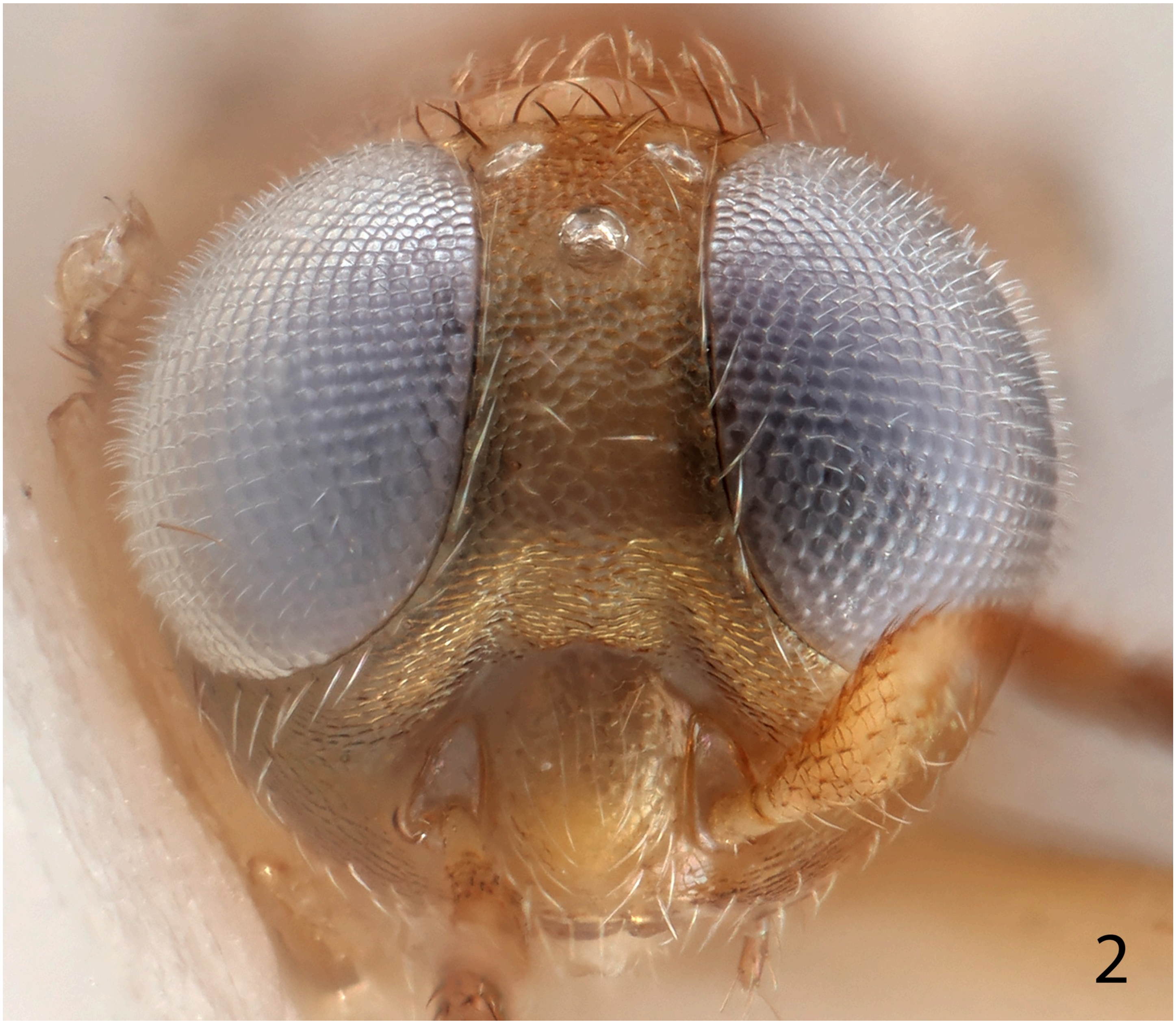

Fig 2. ***Ooencyrtus pitosina*** **holotype female head.**

https://doi.org/10.1371/journal.pone.0288306.g002

different focal depths, were taken of each body part to be illustrated. Each series of images was stacked and merged into a single in-focus image using Helicon Focus ©. Where possible, extraneous micro artifacts (dust, etc.) were removed physically before imaging or the images were digitally cleaned and enhanced using PhotoShop©.

DNA sequencing

Genomic DNA extraction was undertaken from 10 specimens using the protocol in Polaszek *et al.* [10] and Cruaud *et al.* [11], which leaves the sclerotized parts of the specimen intact.

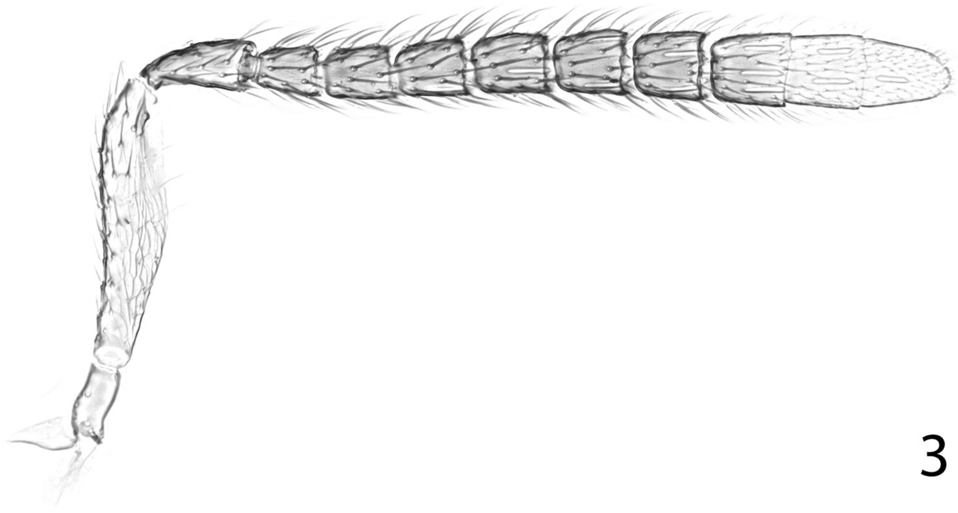

3

Fig 3. ***Ooencyrtus pitosina*** **paratype female, antenna.**

https://doi.org/10.1371/journal.pone.0288306.g003

Specimens were then critical point dried and card-mounted, with selected individuals then dissected and mounted in Canada balsam on microscope slides.

As the Folmer primer pair LCO1490/ HCO2198 [12] does not perform well in many chalcid wasp taxa [13–15], especially in those with suboptimal DNA extracts [16], to get the full-length Cytochrome c Oxidase I (COI) barcode region, the internal primers MChaR1 and MChaF1 [16] were used in combination with the Folmer primers. The 28S D2 fragment was amplified with the primers D23F [17] and 28Sb [18, 19]. The PCR reactions and sequencing were carried out as described in [16]. Forward and reverse sequences were assembled and corrected using Pregap4 and Gap4 software in the Staden package [20]. The p-distance between sequences was computed in MEGA 11 [21]. In our BLAST searches we favoured identity over max score, the last one being influenced by query cover. The sequences have been deposited in GenBank under accession numbers OQ648163–OQ648167 (COI) and OQ650244–OQ650251 (28S), respectively.

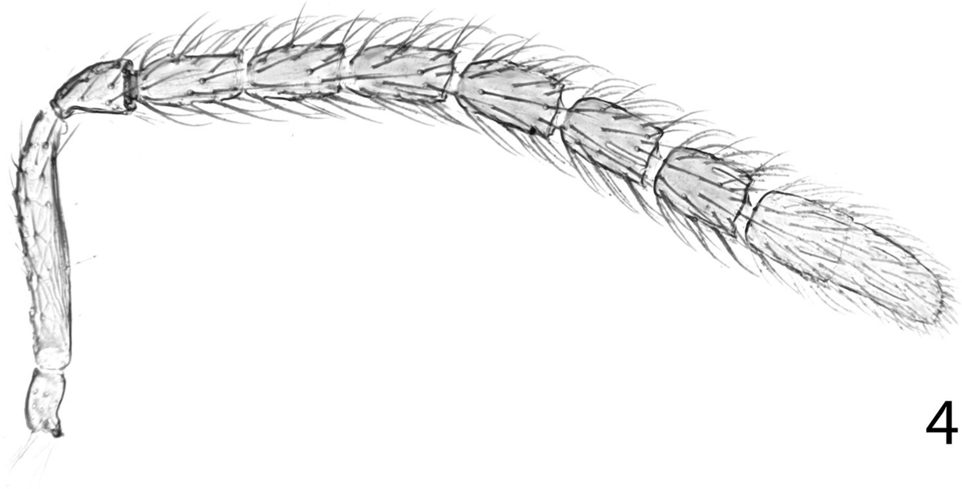

4

Fig 4. ***Ooencyrtus pitosina*** **paratype male, antenna.**

https://doi.org/10.1371/journal.pone.0288306.g004

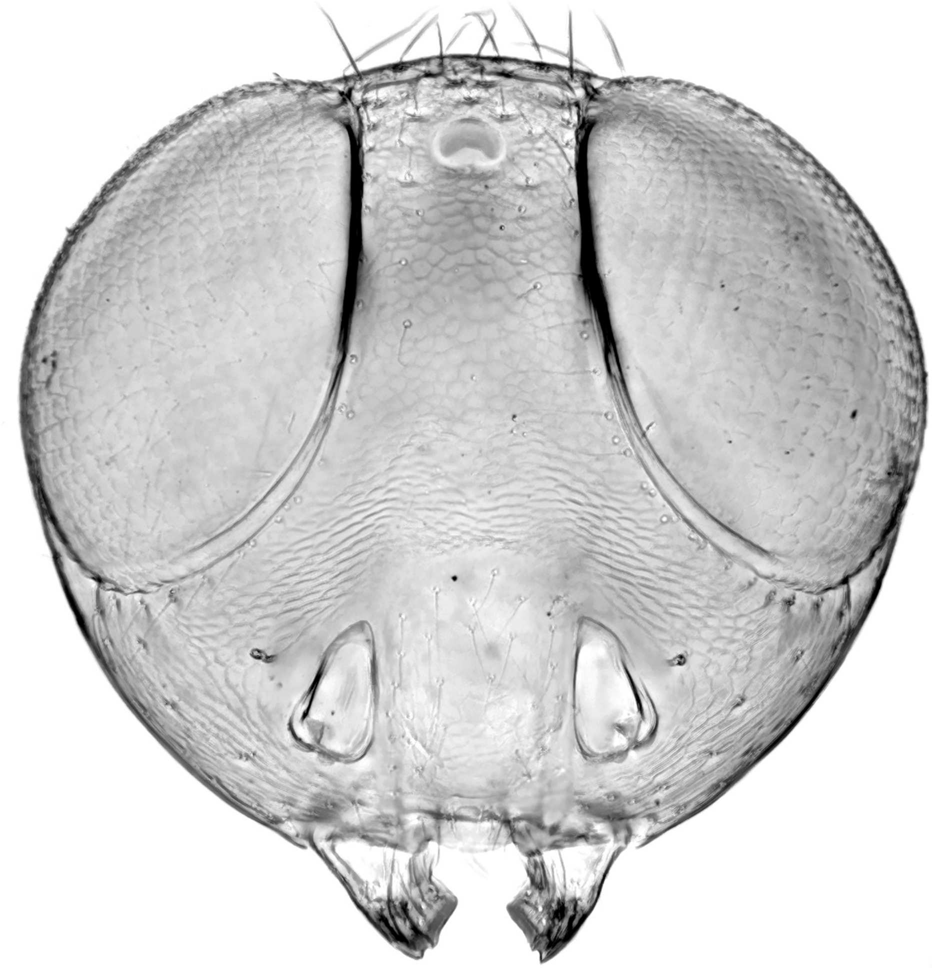

5

Fig 5. ***Ooencyrtus pitosina*** **paratype female head.**

https://doi.org/10.1371/journal.pone.0288306.g005

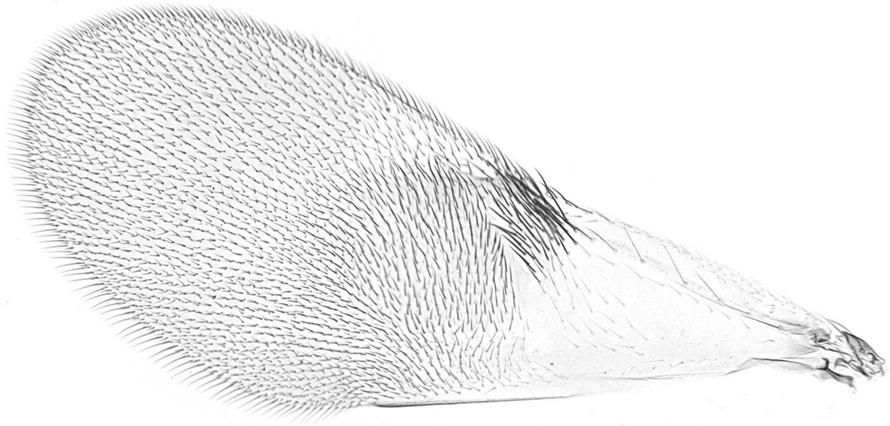

6

Fig 6. ***Ooencyrtus pitosina*** **paratype female, dorsal mesosoma.**

https://doi.org/10.1371/journal.pone.0288306.g006

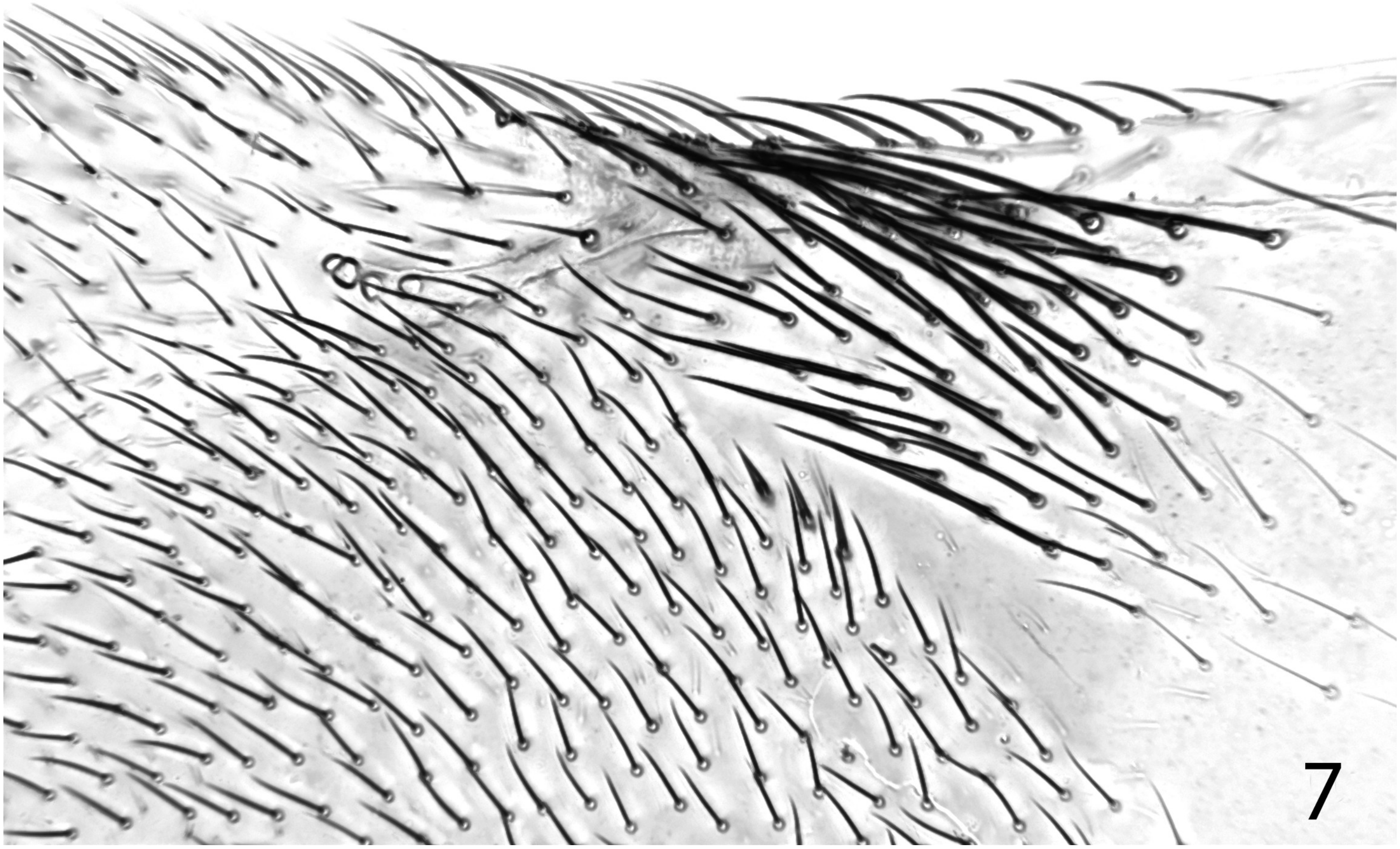

Fig 7. ***Ooencyrtus pitosina*** **paratype female, fore wing.**

https://doi.org/10.1371/journal.pone.0288306.g007

Nomenclatural acts

The electronic edition of this article conforms to the requirements of the amended International Code of Zoological Nomenclature, and hence the new names contained herein are available under the Code from the electronic edition of this article. This published work and the nomenclatural acts it contains have been registered in ZooBank, the online registration system for the ICZN. The ZooBank LSIDs (Life Science Identifiers) can be resolved and the associated information viewed through any standard web browser by appending the LSID to the prefix http://zoobank.org/. The LSID for this publication is: urn:lsid:zoobank.org:pub:94EC4-C18-EEFD-49F6-B4CB-624574A5ABAF

The electronic edition of this work was published in a journal with an ISSN and has been archived and is available from the following digital repositories: PubMed Central, LOCKSS.

Results

Description

Ooencyrtus **Ashmead.** *Ooencyrtus* Ashmead, 1900:381–382 [22]. Type species: *Encyrtus clisiocampae* Ashmead, by original designation.

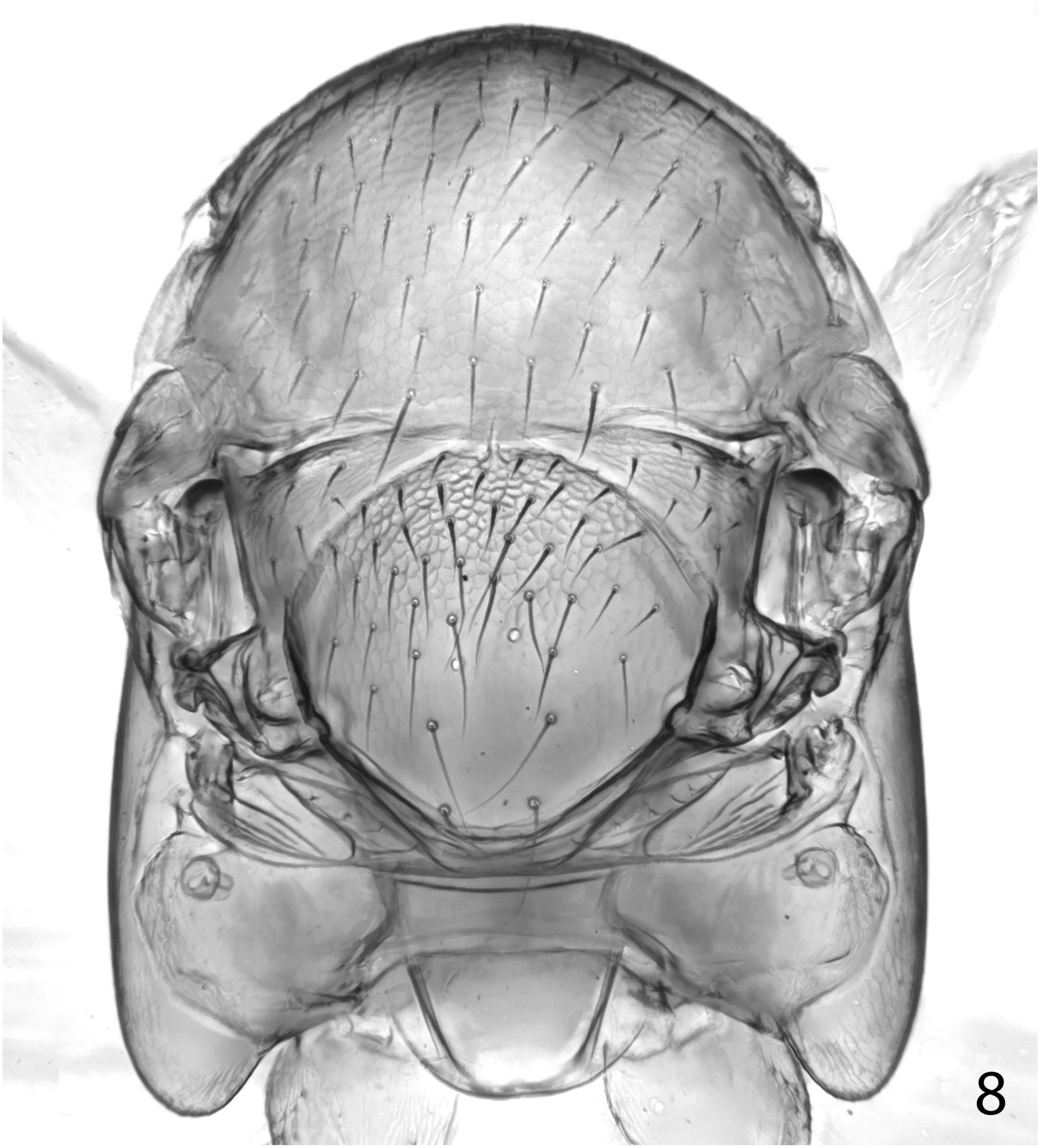

Fig 8. ***Ooencyrtus pitosina*** **paratype female, detail of fore wing.**

https://doi.org/10.1371/journal.pone.0288306.g008

For a complete synonymy list see [23]

Diagnosis. Female (Fig 1). Length about 0.5–2.1mm. Moderately robust, rarely elongate; head and thorax usually dark brown with a metallic sheen, rarely yellow or orange; gaster varying from yellow or orange to dark brown with a metallic sheen; fore wing hyaline, rarely conspicuously infuscate; legs completely orange to brown.

Head (Figs 2, 5) with frontovertex varying from less than one-fifth to about two-fifths head width; occipital margin usually rounded; eye often overreaching occipital margin; antennal torulus separated from mouth margin by about its own length, dorsal margin usually well below lower eye margin; antenna (Fig 3) with funicle 6-segmented; clava 3-segmented, rarely solid or 2-segmented, apex usually rounded; mandible variable, usually with one or two teeth and a truncation, sometimes with 3 acute teeth, rarely with only 2 teeth; palp formula 4–3.

Mesoscutum (Fig 6) with notaular lines absent; axillae narrowly meeting, clearly overlapped medially by posterior margin of mesoscutum; mesopleuron enlarged so that posteriorly it touches base of gaster, in lateral view clearly separating metapleuron and propodeum from hind coxa; fore wing usually fully developed; marginal vein usually more or less punctiform very rarely as much as 4X as long as broad; postmarginal vein mostly very short, sometimes absent, rarely as long as stigmal vein; propodeum with not more than 4 or 5 setae outside spiracle.

Gaster mostly about as long as thorax, occasionally longer than head and thorax combined; paratergites absent; hypopygium not reaching apex of gaster; ovipositor varying from hidden to strongly exserted; gonstylus freely articulated with second valvifer.

Male. Length about 0.6–1.7mm. Sexual dimorphism moderate to extreme, usually similar to female except for structure of antenna and genitalia; sometimes males with conspicuously deeper sculpture and brighter and more metallic than female, rarely thoracic structure very different from that of female; head with torulus separated from mouth margin by at least its own length, lower margin at least about level with lower eye margin; antenna (Fig 4) with all funicle segments longer than broad and clothed in long setae that are generally at least as long as diameter of segments; clava solid, rarely two-segmented; phallobase fairly slender, parameres short, about one-third to half-length of digitus, with a single apical seta; digitus about 2.5× as long as broad with one apical tooth; aedeagus slender, about half to three-quarters as long as mid tibia, with apex acute, rounded or truncate.

Distribution. Cosmopolitan.

Hosts. Most *Ooencyrtus* are primary parasitoids of the eggs of Hemiptera or Lepidoptera, but some species are known to attack lac insects (Hemiptera: Kerriidae), the prepupae of Lepidoptera, braconid (Hymenoptera) primary parasitoids of caterpillars, nymphal stages of Aphididae (Hemiptera), immature stages of Syrphidae (Diptera), pupae of Chloropidae (Diptera), immature stages of Dryinidae (Hymenoptera) attacking auchenorrhynchous Hemiptera, or Coccinellidae (Coleoptera) feeding on aphids, see [23–27].

Identification. [28, 29] catalogues of Nearctic species; [23, 30, 31] Neotropical species; [27] Palaearctic species; [24] Afrotropical species; [25, 26] Indian species; [32] Indo-Pacific species.

Ooencyrtus pitosina **Polaszek, Noyes & Fusu sp. n..** urn:lsid:zoobank.org:act:B11645AD-7830-4028-B938-539F2AE60812

Figs 1–8

Morphology. **Female** (holotype) length: 1.21mm, including ovipositor; 1.16mm, excluding ovipositor.

Head (Fig 2) pale orange with weak brassy reflections, occiput with a small brown mark above foramen; 8 brown setae along occipital margin, 1 behind each posterior ocellus; 4 or 5 translucent pale setae in ocellar area, 8 between anterior ocellus and top of scrobes and a line

along inner eye margin, about 25 on interantennal prominence and several short, inconspicuous setae on gena; antenna with radicle and scape very pale orange; pedicel mostly pale orange-brown, very pale orange ventrally; funicle dark brown; clava very pale yellow, basal segment proximally dark brown; thorax pale orange, neck of pronotum mixed brown; mesoscutum with a slight brassy sheen; tegula very pale yellow with weak coppery reflections; axilla slightly dusky with a slight brassy sheen; scutellum dusky, a little darker than axilla and medially weakly coppery, apical one-third or so with a slight, distinct metallic green and golden sheen; axillula and metanotum laterally dark brown, metanotum medially pale orange; prepectus pale orange; mesopleuron darker orange; prosternum pale orange; mesosternum orange; fore coxa very pale orange, fore leg otherwise yellow, extreme apex of tarsus brown; mid coxa pale orange, mid femur yellow, tibia white, apex and spur yellow, tarsus yellow, extreme apex brown; hind coxa pale orange, hind femur pale orange, a little dusky towards apex dorsally, tibia narrowly pale yellow at base, otherwise dark brown, tarsus yellow; fore wing hyaline with a circular, infuscate area below venation in middle one-third from marginal vein to posterior margin; submarginal vein white, dusky basally; marginal, postmarginal vein and stigmal veins pale orange; propodeum slightly dusky pale orange; about 10 translucent, silvery setae on side near spiracle; gaster with TI dorsally white, side pale orange; venter of gaster pale orange proximally; TII to syntergum of gaster dark brown; gaster with a slight brassy sheen with metallic green and coppery purple reflections, darker areas with a stronger sheen; visible part of gonostylus pale orange.

Head with occipital margin weakly rounded, almost sharp, eyes slightly overreaching occipital margin and clothed in conspicuous, dense, pale setae, each slightly longer than diameter of an eye facet; ocelli forming an angle of about 65˚; frontovertex with very fine, polygonally reticulate sculpture of mesh size slightly less than diameter of eye facet; temple and gena with longitudinally elongate, polygonally reticulate sculpture; interantennal prominence with similar sculpture to frontovertex, but shallower; scrobes moderately deep, ∩-shaped, margins rounded, interantennal prominence dorsally rounded; antennal torulus separated from mouth margin by about its own length. Antenna with funiculars longer than wide except F6 about as long as wide. Relative measurements: HW 88.5, FV 20.5, FVL 51, POL 9.0, OOL 1.0, OCL 6.5, AOL 9, EL 54, EW 49, MS 33.5, SL 39, SW 10.

Thorax with polygonally reticulate sculpture on mesoscutum that is of slightly larger mesh than that on frontovertex and a little shallower; scutellum medially in basal half with very similar sculpture to frontovertex, laterally and posteriorly of slightly larger mesh and a little more shiny; mid tibial spur slightly shorter than basitarsus; fore wing venation and setation as in Figs 7 and 8; marginal vein about 2.5× as long as broad; postmarginal vein about 0.4× as long as stigmal vein. Relative measurements: FWL 204, FWW 78.5, HWL 135, HWW 33.

Gaster slightly shorter than thorax; hypopygium reaching about 0.4× along gaster; syntergum about 0.8× as long as mid tibia, apex rounded; ovipositor slightly exserted, the exserted part about 0.4× as long as mid tibial spur.

Paratype. Funicle with linear sensilla on F2–F6; mandible with one short, acute tooth and broad, very slightly convex truncation. Relative measurements: OV 111, GS 26.5 [MT 91.5].

Variation. The overall length of the female varies from 0.93 to 1.46mm; the pronotum immediately in front of mesothoracic spiracle may be marked dark brown, the posterior half of the scutellum may be orange-brown, the base of the hind tibia may be white and the head varies from 3.8 to 4.4× as wide as frontovertex.

Male (length 0.66–1.16mm): very similar to female but for slightly wider frontovertex, structure and colour of antenna and structure of genitalia. Head about 2.7–3.4× as wide as frontovertex; antenna with funicle dusky orange; funicle with all segments longer than broad; clava 2-segmented, completely white; phallobase with digiti about 3.6× as long as broad and

each with a single apical tooth, parameres developed, each with a basal and a subapical seta; aedeagus slender with apex strongly acute, Relative measurements: MT 61.5, AL 36.5.

Molecular analysis. We obtained five full length DNA barcodes (COI) and eight 28S sequences 858 bp in length (not all 10 extracted specimens had high quality DNA). The COI sequences revealed three haplotypes differing at two variable sites. The 28S sequences were all identical. The paucity of DNA sequences for *Ooencyrtus* species in GenBank or in Barcode of Life Data System (BOLD) have precluded the need for any phylogenetic analyses. A GenBank BLAST of our COI sequences resulted in *Ooencyrtus plautus* Huang & Noyes (assuming correct identification), from a recently published complete mitochondrial genome study [33] as the top hit. The following 10 similar sequences are as follows: two of *Ooencyrtus nezarae* from [34], five of *Metaphycus flavus*, and three of Encyrtidae sp. or Hymenoptera sp. The last three sequences (KJ208550.1, KY831595.1, KY845087.1) are derived from BOLD and based on their images can be identified as *Ooencyrtus* sp. and *Lamennaisia* sp. Even *O. plautus*, the most similar species present in GenBank, is 13.9–14.1% divergent from *Ooencyrtus pitosina*.

Using BLAST our 28S sequences were found to have a 90.4% identity with *Ooencyrtus* sp. 1 YZZ-2014 (accession KF850145.1), a sequence derived from a paratype of *O. protohermesis* Zhang & Zhang [35]. The *p*-distance between this species and *O. pitosina* is 7.9%.

Hosts. A gregarious parasitoid of the eggs of *Papilio godeffroyi* on leaves of *Micromelum minutum*.

Distribution. American Samoa.

Material examined. Holotype ♀: AMERICAN SAMOA, Tutuila Island, Afono, ex *Papilio godeffroyi* egg on *Micromelum minutum* leaf, ASPG0824, Host coll 16.i.2014 (M. Schmaedick). Paratypes: AMERICAN SAMOA, 2♀, 2♂, Tutuila Island, Vatia, ex *Papilio godeffroyi* egg on *Micromelum minutum* leaf, Host coll. 9.i.2013 (M. Schmaedick); 12♀, 4♂, Tutuila Isl., Vatia, ex egg of *Papilio godeffroyi* on *Micromelum minutum*, coll. 21–22.i.2022 (N. Leifi); 4♀, 3♂, Tutuila Island, Masausi, ex *Papilio godeffroyi* egg on *Micromelum minutum* leaf, Host coll. 11.i.2013 (M. Schmaedick); 2♀, Tutuila Island, Aoa, ex *Papilio godeffroyi* egg on *Micromelum minutum* leaf, ASPG0081, Host coll. 24.vii.2013 (M. Schmaedick); 46♀, 26♂, Tutuila Isl., Afono, ex egg of *Papilio godeffroyi* on *Micromelum minutum*, Host coll. 19.xii.2013, 16.i.2014, 21.ii.2014, 13.vi.2014, 19.vi.2014 (M. Schmaedick); 47♀, 18♂, Tutuila Isl., Maloata, ex egg of *Papilio godeffroyi* on *Micromelum minutum*, Host coll. 24.xii.2013, 30.i.2014, 21.ii.2014, 7.iii.2014, 25.iv.2014, 23.v.2014 (M. Schmaedick); 7♀, 1♂, Tutuila Isl., Malaeimi, ex egg of *Papilio godeffroyi* on *Micromelum minutum*, Host coll. 18–20.i.2022 (N. Leifi). Holotype in NHMUK, paratypes in AICF, BPBM, NHMUK and USNM.

Etymology. "pito" = "tip" and "sina" ="white" in Samoan, referring to the pale-tipped antenna. Noun in apposition.

Comments. *Ooencyrtus pitosina* can be distinguished from all known species of the genus by the unique combination of the generally orange colour of the body, mandible with a small tooth and broad truncation, infuscate fore wing and elongate marginal vein of the fore wing. In the key to Indo-Pacific species [32] it runs easily to *Ooencyrtus lucina* Huang & Noyes at couplet 52 which shares a similar general coloration. No almost completely orange species of *Ooencyrtus* are known in other parts of the world. The two species can be distinguished in both sexes by the relative length of the marginal vein which is about 2.5× as long as broad in *pitosina* and punctiform in *lucina*. In addition, females of *pitosina* have the frontovertex about one-quarter head width, F1 smaller than remaining funicle segments which are subequal in size, the hind tibia dark brown, fore wing conspicuously infuscate and ovipositor slightly exserted. In females of *lucina* the fontovertex is one-third head width, F1–F2 conspicuously smaller than remainder which are not subequal, the hind tibia is yellow, the fore wing is completely hyaline, and the ovipositor is not exserted. On the other hand, males of *pitosina*

have the head orange, clava completely white, frontovertex about one-third head width, fore wings weakly infuscate and hind tibia dark brown, whilst in males of *lucina* the head has a dull dark green and purple sheen, the basal half of the clava is brown, the frontovertex is about half head width and the fore wing is completely hyaline. In the key to Indian species [25] *pitosina* runs to couplet 62 and clearly differs from any of the species included after that point because none is orange, and all have hyaline wings. Other regional comprehensive keys to the species of *Ooencyrtus*—Costa Rica [23], southern Africa [24] and Palaearctic [27] do not include any species that are yellow or orange in colour, and very few species included in those treatments have a marginal vein that is more than 1.5× as long as broad. The only group of species currently placed in *Ooencyrtus* that have a completely orange (or yellow) head and thorax and long marginal vein are those species that are known to parasitize lac insects (Kerriidae). Two of these species also have the fore wing weakly partially infuscate, viz: *O. kerriae* Hayat and *O. paratachardinae* Hayat, Schroer & Pemberton. These species are superficially somewhat similar to *pitosina* but differ in the mandible having three uniform acute teeth.

Because of the unusually elongate marginal vein of the fore wing, we considered it worth comparing *Ooencyrtus pitosina* with species currently placed in similar or closely related genera of Encyrtidae. The most similar of these is *Hengata spinosa* Noyes & Hayat [36] described from Sulawesi (Indonesia) with an unknown biology. The species are superficially very similar in that they are both generally orange in colour and have similar antennae. However, *Ooencyrtus pitosina* differs from *Hengata* in lacking a median longitudinal ridge on the mesoscutum, having a mandible with one short tooth and a very broad truncation and the male scape lacking a spine on its ventral margin. In *Hengata* the mesoscutum has a median, longitudinal, posterior ridge, the mandible is tridentate with the upper tooth truncate and the male scape has an elongate, distinct spine on its ventral margin. Further to this in *Ooencyrtus pitosina* the clava is proximally brown and the fore wing strongly infuscate medially whereas in *Hengata spinosa* the clava is completely white and the fore wing hyaline.

Discussion

It appears that *O pitosina* is not closely related to any species with sequences currently deposited in Genbank. Neither the unidentified species of *Ooencyrtus* nor *O. plautus* or *O. nezarae* (the closest matches based on COI) bear any resemblance to *O. pitosina*. The most similar species based on 28S, *O. protohermesis*, has completely yellow legs, a yellow acropleuron and possibly a yellow prosternum [35] but otherwise they are dissimilar. The fact that we found three haplotypes out of five COI sequences indicates that *O. pitosina* might be a long-established species in American Samoa, though a recent human-mediated introduction cannot be excluded. Accidentally introduced Hymenoptera species frequently have reduced genetic diversity resulting from the small number of colonising individuals (usually only one haplotype is present in the recently colonised areas) [37–39].

Acknowledgments

The authors thank Niela Leifi for technical assistance in the field, and the American Samoa Community College Samoan Studies Institute for assistance with the species name. Field and laboratory studies in American Samoa were supported by the Natural Resources Protection Program, a collaboration between the U.S. Geological Survey (USGS) and the National Park Service, the USGS Wildlife Program, and the U.S. Department of Agriculture National Institute of Food and Agriculture Hatch Program. Any use of trade, firm, or product names is for descriptive purposes only and does not imply endorsement by the U.S. Government

Author Contributions

Conceptualization: Andrew Polaszek, Mark A. Schmaedick, Robert W. Peck, Paul C. Banko.

Data curation: Andrew Polaszek, Mark A. Schmaedick, Robert W. Peck, Paul C. Banko, Lucian Fusu.

Formal analysis: Andrew Polaszek, Robert W. Peck, Paul C. Banko, Lucian Fusu.

Funding acquisition: Andrew Polaszek, Mark A. Schmaedick, Robert W. Peck, Paul C. Banko.

Investigation: Elena B. Lugli, Mark A. Schmaedick, Robert W. Peck, Paul C. Banko, Lucian Fusu.

Methodology: Andrew Polaszek, Elena B. Lugli, Mark A. Schmaedick, Robert W. Peck, Paul C. Banko, Lucian Fusu.

Resources: Mark A. Schmaedick, Robert W. Peck, Paul C. Banko.

Supervision: Andrew Polaszek.

Validation: Mark A. Schmaedick.

Visualization: Mark A. Schmaedick.

Writing – original draft: Andrew Polaszek, John S. Noyes, Elena B. Lugli, Mark A. Schmaedick, Robert W. Peck, Paul C. Banko, Lucian Fusu.

Writing – review & editing: Andrew Polaszek, John S. Noyes, Elena B. Lugli, Mark A. Schmaedick, Robert W. Peck, Paul C. Banko, Lucian Fusu.

References

1. Tennent WJ. A checklist of the butterflies of Melanesia, Micronesian, Polynesia and some adjacent areas. Zootaxa 2006; 1178(1): 1–209. https://doi.org/10.11646/zootaxa.1178.1.1
2. Patrick B, Patrick H. Butterflies of the South Pacific. Dunedin: Otago University Press; 2012.
3. Patrick B, Edwards E, Böhm M. Papilio godeffroyi, Samoan swallowtail [Internet]. The IUCN Red List of Threatened Species. 2018 [cited 2023 Mar 20]: Available from: e.T122530997A122603026. https://doi.org/10.2305/IUCN.UK.2018-2.RLTS.T122530997A122603026.en
4. Whistler WA. Rainforest trees of Samoa: a guide to the common lowland and foothill forest trees of the Samoan Archipelago. Isle Botanica, Honolulu, Hawaii, USA. 2004.
5. Mathew GF. Life history of three species of Western Pacific Rhopalocera. Trans. Ent. Soc. Lond. 1885 Sep (Pt V):357–368. https://doi.org/10.1111/j.1365-2311.1885.tb00890.x
6. Patrick B, Chandra V. Papilio schmeltzi [Internet]. The IUCN Red List of Threatened Species. 2019 [cited 2023 Mar 20]: Available from: e.T160879A859235. https://dx.doi.org/10.2305/IUCN.UK.2019-3.RLTS.T160879A859235.en.
7. Banko PC, Peck RW, Schmaedick MA, Miles AC, Leifi N, Brinck KW. Reproductive response of the Samoan swallowtail butterfly to variability in host plant and habitat characteristics. Ecosphere (*in press*).
8. Banko PC, Schmaedick MA, Peck RW, Miles AC, Leifi N. Reproductive demography and egg parasitism of the Samoan swallowtail butterfly. Ecosphere (*in revision*).
9. Noyes JS *Encyrtidae of Costa Rica (Hymenoptera*: *Chalcidoidea)*, *2*. Metaphycus *and related genera*, *parasitoids of scale insects (Coccoidea) and whiteflies (Aleyrodidae)*. Mem. American Ent. Inst. 73: 1–459. 2004
10. Polaszek A, Ayshford T, Yahya BE, Fusu L *Wallaceaphytis*: an unusual new genus of parasitoid wasp (Hymenoptera: Aphelinidae) from Borneo. J Nat Hist, 48 (19–20), 1111–1123. 2014 https://doi.org/10.1080/00222933.2013.852264
11. Cruaud A, Nidelet S, Arnal P, Weber A, Fusu L, Gumovsky A, et al. (2019) Optimised DNA extraction and library preparation for minute arthropods: application to target enrichment in chalcid wasps used for biocontrol. Mol. Ecol. Res. 19:702–710. https://doi.org/:10.1111/1755-0998.13006

12. Folmer O, Black M, Hoeh W, Lutz R, Vrijenhoek R DNA primers for amplification of mitochondrial cytochrome c oxidase subunit I from diverse metazoan invertebrates. Mol. Mar. Biol. Biotech. 1994; 3: 294–299.

13. Kaartinen R, Stone GN, Hearn J, Lohse K & Roslin T Revealing secret liaisons: DNA barcoding changes our understanding of food webs. Ecol. Ent. 35 (5), 623–638. 2010. https://doi.org/10.1111/j.1365-2311.2010.01224.x

14. Lohse K, Sharanowski B, Blaxter M, Nicholls JA & Stone GN Developing EPIC markers for chalcidoid Hymenoptera from EST and genomic data. Mol. Ecol. Res. 11 (3), 521–529. 2011 https://doi.org/10.1111/j.1755-0998.2010.02956.x

15. Baur H, Kranz-Baltensperger Y, Cruaud A, Rasplus J-Y, Timokhov AV & Gokhman VE Morphometric analysis and taxonomic revision of *Anisopteromalus* Ruschka (Hymenoptera: Chalcidoidea: Pteromalidae)–an integrative approach. Syst. Ent. 39 (4), 691–709 2014. https://doi.org/10.1111/syen.12081

16. Fusu L, Polaszek A Description, DNA barcoding and phylogenetic placement of a remarkable new species of *Eopelma* (Hymenoptera: Eupelmidae) from Borneo. Zootaxa 4263 (3): 557–566; 2017. https://doi.org/10.11646/zootaxa.4263.3.7

17. Park JK, O'Foighil D Sphaeriid and corbiculid clams represent separate heterodont bivalve radiations into freshwater environments. Mol. Phyl. Evol., 14(1), 75–88. 2000. https://doi.org/10.1006/mpev.1999.0691 PMID: 10631043

18. Whiting MF, Carpenter JC, Wheeler QD, Wheeler WC. The Strepsiptera problem: phylogeny of the holometabolous insect orders inferred from 18S and 28S ribosomal DNA sequences and morphology. Syst. Biol. 46(1), 1–68. 1997. https://doi.org/10.1093/sysbio/46.1.1 PMID: 11975347

19. Nunn GB, Theisen BF, Christensen B, Arctander P. Simplicity correlated size growth of the nuclear 28S ribosomal RNA D3 expansion segment in the crustacean order Isopoda. J. Mol. Evol. 42: 211–223. 1996. https://doi.org/10.1007/BF02198847 PMID: 8919873

20. Staden R, Judge DP, Bonfield JK. Sequence assembly and finishing methods. In: Baxevanis AD, Ouellette BFF, editors. Bioinformatics: a practical guide to the analysis of genes and proteins. Methods of Biochemical Analysis 43. New York: John Wiley & Sons Inc.; 2001. p. 303–322. https://doi.org/10.1002/0471223921.ch13

21. Tamura K, Stecher G, Kumar S. MEGA11: Molecular Evolutionary Genetics Analysis Version 11. Mol. Biol. Evol. 2021; 38(7):3022–3027. https://doi.org/10.1093/molbev/msab120 PMID: 33892491

22. Ashmead W.H. 1900. On the genera of chalcid-flies belonging to the subfamily Encyrtinae. Proc. United States Natl. Mus. 22:323–412

23. Noyes J.S. 2010 Encyrtidae of Costa Rica (Hymenoptera: Chalcidoidea), 3. Subfamily Encyrtinae: Encyrtini, Echthroplexiellini, Discodini, Oobiini and Ixodiphagini, parasitoids associated with bugs (Hemiptera), insect eggs (Hemiptera, Lepidoptera, Coleoptera, Neuroptera) and ticks (Acari). Memoirs of the American Entomological Institute 84:848pp

24. Prinsloo G.L. 1987, A revision of the genus *Ooencyrtus* Ashmead (Hymenoptera: Encyrtidae) in subsaharan Africa. Entomology Memoir, Department of Agriculture and Water Supply, Republic of South Africa 67:1–46

25. Hayat M.; Zeya S.B.; Veenakumari K. 2017a, The species of *Ooencyrtus* Ashmead (Hymenoptera: Encyrtidae) from India—1. Journal of Insect Systematics 3(1–2):42–58

26. Hayat M.; Zeya S.B.; Veenakumari K. 2017b, The species of *Ooencyrtus* Ashmead (Hymenoptera: Encyrtidae) from India—2. J. Insect Syst. 4(1):18–83

27. Trjapitzin VA. 1989, Parasitic Hymenoptera of the Fam. Encyrtidae of Palaearctics. Opredeliteli po Faune SSSR 158:489 pp. Zoologicheskii Institut Akademii Nauk SSSR, Leningrad

28. Peck O. 1963, A catalogue of the Nearctic Chalcidoidea (Insecta; Hymenoptera). Canadian Ent. (Supplement) 30:1–1092

29. Gordh G. 1979, Family Encyrtidae. Catalog of Hymenoptera in America North of Mexico 1:890–967 (Eds: Krombein, K.V.; Hurd, P.D. jr; Smith D.R.; Burks, B.D.) Washington, D.C.

30. Noyes J.S. 1985, A review of the Neotropical species of *Ooencyrtus* Ashmead, 1900 (Hymenoptera: Encyrtidae). J. Nat. History 19:533–554

31. De Santis L. 1988, Calcidoides nuevos de la Republica Argentina y Chile (Insecta, Hymenoptera). Revista Chilena de Entomología 16:41–48

32. Huang D.W.; Noyes J.S. 1994, A revision of the Indo-Pacific species of *Ooencyrtus* (Hymenoptera: Encyrtidae), parasitoids of the immature stages of economically important insect species (mainly Hemiptera and Lepidoptera). Bulletin of the Natural History Museum (Entomology Series) 63(1):136pp

33. Xing ZP, Liang X, Wang X, Hu HY, Huang YX. Novel gene rearrangement pattern in mitochondrial genome of *Ooencyrtus plautus* Huang & Noyes, 1994: new gene order in Encyrtidae (Hymenoptera, Chalcidoidea). ZooKeys. 2022; 1124:1–21. https://doi.org/10.3897/zookeys.1124.83811

34. Ademokoya B, Balusu R, Ray C, Mottern J, Fadamiro H. The first record of *Ooencyrtus nezarae* (Hymenoptera: Encyrtidae) on Kudzu Bug (Hemiptera: Plataspidae) in North America. J. Insect Sci. 2018; 18 (1): 8. https://doi.org/10.1093/jisesa/iex105

35. Zhang X, Zhang Y-Z, Wang Y, Chen F-Q, Yu F, Zhou Q-S. Description of three new species of *Ooencyrtus* (Hymenoptera: Encyrtidae) from China. Zootaxa. 2014; 3790(3):451–465 http://dx.doi.org/10.11646/zootaxa.3790.3.4

36. Noyes JS, Hayat M Oriental mealybug parasitoids of the Anagyrini (Hymenoptera: Encyrtidae) viii +554 pp. CAB International, UK. 1994.

37. Sabbatini Peverieri G., Mitroiu M. D., Bon M. C., Balusu R., Benvenuto L., Bernardinelli I., et al. (2019). Surveys of stink bug egg parasitism in Asia, Europe and North America, morphological taxonomy, and molecular analysis reveal the Holarctic distribution of *Acroclisoides sinicus* (Huang & Liao) (Hymenoptera, Pteromalidae). J. Hymenopt. Res. 74, 123–151. https://doi.org/10.3897/jhr.74.46701

38. Stahl J., Tortorici F., Pontini M., Bon M. C., Hoelmer K., Marazzi C., et al. (2019). First discovery of adventive populations of *Trissolcus japonicus* in Europe. J. Pest. Sci. 92, 371–379. https://doi.org/10.1007/s10340-018-1061-2

39. Dillane E., Hayden R., O'Hanlon A., Butler F., & Harrison S. (2022). The first recorded occurrence of the Asian hornet (*Vespa velutina*) in Ireland, genetic evidence for a continued single invasion across Europe. J. Hymenopt. Res. 93, 131–138. https://doi.org/10.3897/jhr.93.91209
